# Kinetics and Thermodynamics of Uranium (VI) Adsorption onto Humic Acid Derived from Leonardite

**DOI:** 10.3390/ijerph16091552

**Published:** 2019-05-02

**Authors:** Fande Meng, Guodong Yuan, Steven L. Larson, John H. Ballard, Jeremy R. White, Zikri Arslan, Fengxiang X. Han

**Affiliations:** 1 School of Environmental and Chemical Engineering, Zhaoqing University, Zhaoqing 526061, China; fdmeng01@foxmail.com; 2 Department of Chemistry and Biochemistry, Jackson State University, Jackson, MS 39217, USA; jeremy.r.white@students.jsums.edu (J.R.W.); zikri.arslan@jsums.edu (Z.A.); 3 College of Resource and Environment, Anhui Science and Technology University, Chuzhou 233100, China; 4 U.S. Army Engineer Research and Development Center, Vicksburg, MS 39180-6199, USA; Steven.L.Larson@usace.army.mil (S.L.L.); John.H.Ballard@usace.army.mil (J.H.B.)

**Keywords:** humic acid, uranium (VI), FTIR, adsorption, chemisorption

## Abstract

Humic acid (HA) is well known as an inexpensive and effective adsorbent for heavy metal ions. However, the thermodynamics of uranium (U) adsorption onto HA is not fully understood. This study aimed to understand the kinetics and isotherms of U(VI) adsorption onto HA under different temperatures from acidic water. A leonardite-derived HA was characterized for its ash content, elemental compositions, and acidic functional groups, and used for the removal of U (VI) from acidic aqueous solutions via batch experiments at initial concentrations of 0–100 mg·L^−1^ at 298, 308 and 318 K. ICP-MS was used to determine the U(VI) concentrations in solutions before and after reacting with the HA. The rate and capacity of HA adsorbing U(VI) increased with the temperature. Adsorption kinetic data was best fitted to the pseudo second-order model. This, together with FTIR spectra, indicated a chemisorption of U(VI) by HA. Equilibrium adsorption data was best fitted to the Langmuir and Temkin models. Thermodynamic parameters such as equilibrium constant (K_0_), standard Gibbs free energy (ΔG^0^), standard enthalpy change (ΔH^0^), and standard entropy change (ΔS^0^), indicated that U(VI) adsorption onto HA was endothermic and spontaneous. The co-existence of cations (Cu^2+^, Co^2+^, Cd^2+^ and Pb^2+^) and anions (HPO_4_^2−^ and SO_4_^2−^) reduced U(VI) adsorption. The high propensity and capacity of leonardite-derived HA adsorbing U(VI) suggests that it has the potential for cost-effective removal of U(VI) from acidic contaminated waters.

## 1. Introduction

Uranium (U) is widely but unevenly distributed in soils with an average concentration of 2.6 mg·kg^−1^ [1]. Acid mining drainage is a major source of U release into soil and water environments [2,3]. Naturally occurring U consists of three isotopes: U-238 (99.2739–99.2752%), U-235 (0.7198–0.7202%) and U-234 (0.0050–0.0059%). In oxidizing environments U is usually found in hexavalent form. U accumulation moves up the food chain, and eventually, to human organs and tissues, causing severe damage to kidneys, liver and in extreme cases, death [4]. The World Health Organization and US EPA have set the maximum concentration for U in drinking water at 15 and 30 μg·L^−1^, respectively [5,6].

Adsorption, chemical precipitation, coagulation/flocculation, ultrafiltration and reverse osmosis are common processes used for removing U from wastewaters [7]. Adsorption of U(VI) onto insoluble adsorbents, such as clay minerals, activated carbon, biochar and natural biopolymers, has been investigated [8,9,10,11,12]. Humic acid (HA) is an inexpensive biopolymer with abundant functional groups (carboxylic and phenolic-hydroxyl), and it has been utilized to adsorb heavy metal ions [13].

U(VI) adsorption onto HA has been described with many models, including the pseudo second-order equation [8,14]. The thermodynamics of U(VI) adsorption onto HA, however, is not fully understood. This study aimed to understand the kinetics and isotherms of U(VI) adsorption onto HA under different temperatures from acidic water to reveal the equilibrium time, the mechanisms, and the capacities of U adsorption onto HA.

## 2. Materials and Methods

### 2.1. Materials and Reagents

A leonardite was purchased from Leonardite Products, LLC, in Williston, ND, USA. All reagents used in this study were of analytical grade. Copper nitrate (Cu(NO_3_)_2_), cadmium nitrate (Cd(NO_3_)_2_), cobalt nitrate (Co(NO_3_)_2_), hydrochloric acid (HCl), lead nitrate (Pb(NO_3_)_2_), nitric acid (HNO_3_) and sodium hydroxide (NaOH) were purchased from Thermo Fisher (Waltham, MA, USA). Uranyl nitrate (1%) was purchased from Poly Scientific R&D Corp (Bay Shore, NY, USA). U(VI) solutions were prepared for batch adsorption experiments by successively diluting the aqueous 1% uranyl nitrate with 1 mM sodium nitrate (NaNO_3_) as a background electrolyte [15]. HNO_3_ and NaOH were used for adjusting solution pH.

### 2.2. Preparation and Characterization of HA

HA was extracted from the leonardite with traditional alkaline-acid protocol [8]. Briefly, 25 g leonardite was placed into a Teflon-container with 250 mL 0.1 M NaOH and sonicated for 30 min. After standing overnight, the supernatant was collected. This process was repeated 2 more times for a total of 3 extractions. The collected supernatants were combined, and small aliquots of 6 M HCl was titrated in, while stirring, until the pH was reduced to 2. The suspensions were then centrifuged at 3000 g for 15 min. The precipitates (HA) were washed three times with distilled water and then freeze-dried for later use.

The physical and chemical properties of the leonardite and derived HA were analyzed as follows: Ash content was determined with ignition in a muffle furnace at 800 °C for 4 h under atmospheric condition. Elemental compositions were determined with an elemental analyzer (Vario micro cube, Elementar, Germany) for dried samples at 80 °C. Functional groups were identified with Fourier transform infrared spectroscopy (Spectrum Two, PerkinElmer, Waltham, MA, USA), and acidic functional groups were quantified with the titration method of the International Humic Substances Society [16].

### 2.3. Adsorption Experiments and Data Processing

All adsorption experiments were conducted in duplicates, including blanks and calibration controls. Briefly, 20 mg of HA was weighed into 50 mL plastic centrifuge tubes (Corning, Corning, NY, USA) with 30 mL U solution, and the pH of the suspension was adjusted to 3.0. The tubes were then shaken for 6 h to achieve equilibrium. Then, the tubes were centrifuged, and the supernatants were filtered through a 0.45 μm membrane (Whatman, Little Chalfont, Buckinghamshire, UK) for analysis of U concentration with an ICP-MS (Varian Inc., Palo Alto, CA, USA). The pH at the beginning and end of adsorption experiment was measured by a pH meter (Oakton, Vernon Hills, IL, USA).

U adsorption on the HA was calculated from the difference in concentrations before and after the adsorption. MS-Excel and OriginPro 8.0 (OriginLab, Wellesley Hills, MA, USA) were used for data processing.

## 3. Models

### 3.1. Adsorption Kinetics Models

Parameters obtained from four adsorption models were used to describe the kinetics of U(VI) adsorption onto HA: pseudo first-order model (Equation (1)) was used to describe the adsorption process in solid-liquid system at the initial phase, which corresponds to a diffusion-controlled process [17,18]; pseudo second-order model (Equation (2)) was used to describe whole adsorption process, involving chemisorption in solid-liquid system [18,19]; the Elovich equation (Equation (3)) was used to describe the chemisorption that occurred on heterogeneous solid surface [20,21]; and the intraparticle diffusion model (Equation (4)) was used to determine the intraparticle diffusion rate constant and the boundary resistance [22]. Detailed descriptions on the models and parameters are available in the literature [17,18,19,20,21,22].

(1) $$q_{t}=q_{1}\left(1-e^{-k_{1}t}\right)$$

(2) $$q_{t}=\frac{q_{2}^{2}k_{2}t}{1+q_{2}k_{2}t}\;\;\;\;\;$$

(3) $$q_{t}=\frac{1}{\beta }ln\left(\alpha \beta \right)+\frac{1}{\beta }ln\left(t\right)$$

(4) $$q_{t}=k_{i}t^{0.5}+C$$

### 3.2. Adsorption Isotherm Models

Four adsorption isotherm models were used to describe U distribution between solution and HA at the equilibrium state: the Freundlich model (Equation (5)) describes both monolayer and multilayer adsorption, which is based on heterogeneous adsorption in solid-liquid system [23,24]; the Langmuir model (Equation (6)) quantifies the adsorption capacity [8,25,26]; the Temkin model (Equation (7)) takes U-HA interaction into account and links adsorption energy to the adsorbent surface [27]; and the Dubinin–Radushkevich (D-R) model (Equation (8)) describes adsorption reaction at low concentration ranges on the homogeneous or heterogeneous surface [28].

(5) $$q_{e}=k_{F}C_{e}^{1/n}$$

(6) $$q_{e}=\frac{q_{L}k_{L}C_{e}}{1+k_{L}C_{e}}$$

(7) $$q_{e}=\frac{RT}{b}lnk_{T}+\frac{RT}{b}lnC_{e}$$

(8) $$q_{e}=q_{D}e^{-k_{D}RTln{\left(1+\frac{1}{C_{e}}\right)}^{2}}$$

### 3.3. Thermodynamic Parameters

The thermodynamic parameters are usually used to illustrate adsorption mechanisms and determine the reaction direction, which can be calculated from the thermodynamic equilibrium constant, *K*_0_. The standard Gibbs free energy Δ*G*^0^ (kJ·mol^−1^), standard enthalpy change Δ*H*^0^ (kJ·mol^−1^), and standard entropy change Δ*S*^0^ (J·mol^−1^·K^−1^) were determined from the equations as follows:

(9) $$\Delta G^{0}=-RTlnK_{0}$$

(10) $$lnK_{0}=\frac{\Delta S^{0}}{R}-\frac{\Delta H^{0}}{RT}$$

*K*_0_ can be defined as,

(11) $$K_{0}=\frac{q_{e}}{C_{e}}$$

where *R* is the gas constant (8.314 J·mol^−1^·K^−1^), *T* is the temperature in K, *C_e_* is the equilibrium concentration (mg·L^−1^), and *q_e_* is the amount of adsorption at equilibrium state (mg·g^−1^).

## 4. Results and Discussion

### 4.1. Properties of Adsorbents

The properties of the leonardite and HA are shown in Table 1. HA had lower pH and ash content, but higher C and O contents than leonardite. Both HA and leonardite had abundant acidic functional groups (carboxyl and phenolic-hydroxyl), of which carboxyl groups are considered as the most important for adsorbing metal ions [13].

**Figure 1:**
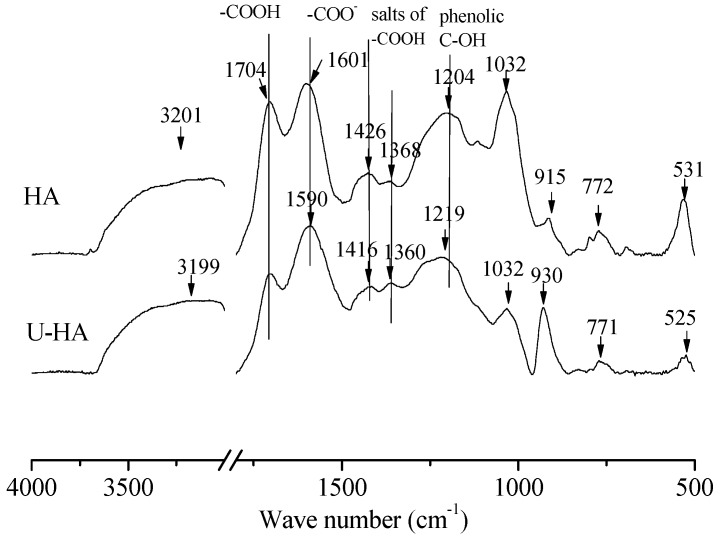
The FTIR spectra of humic acid (HA) before and after reaction with U(VI).

The FTIR spectra of HA (Figure 1) confirmed the existence of oxygen-containing functional groups, as shown at wavenumbers of 3201 cm^−1^ (OH stretch of phenolic-OH), 1704 cm^−1^ (C=C stretch of COOH groups), 1601 cm^−1^ (asymmetric -COO^–^ stretch), 1426 cm^−1^ (symmetric -COO^–^ stretch), 1368 cm^−1^ (salts of -COOH), 1204 cm^−1^ (-C-O stretch and phenolic C-OH) and 1032 cm^−1^ (O-CH_3_ vibrations) [29,30].

### 4.2. Adsorption Kinetics

**Figure 2:**
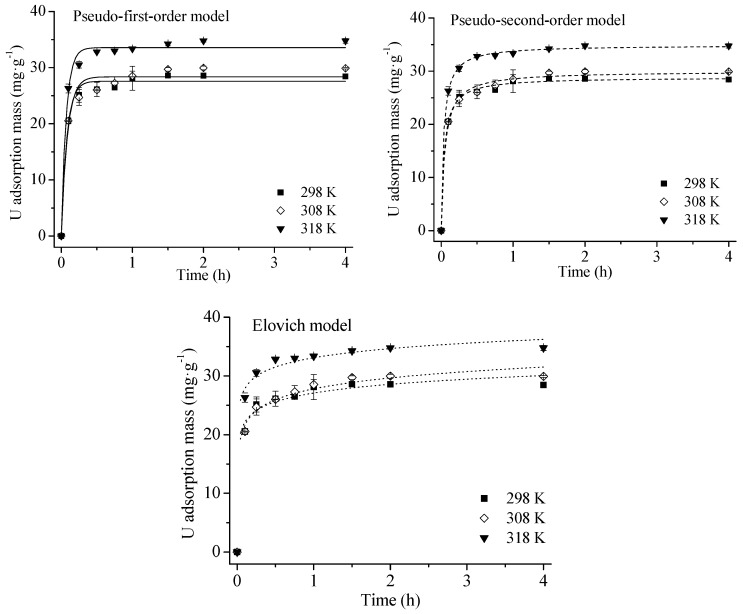
Kinetic models for U adsorption onto HA at different temperatures. Experimental conditions: adsorbent mass: 20 mg; solution volume: 30 mL; U(VI) concentration: 60 mg/L; contact time: 0.1, 0.25, 0.5, 0.75, 1, 1.5, 2 and 4 h; initial pH = 3.0; end pH: 2.70–2.90.

**Figure 3:**
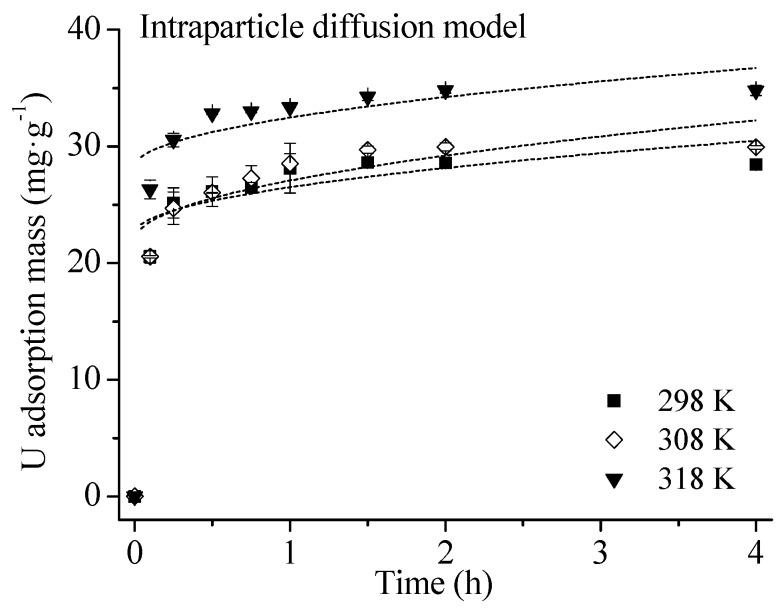
Intraparticle diffusion model for U(VI) adsorption onto HA at different temperatures. Experimental conditions: adsorbent mass: 20 mg; solution volume: 30 mL; U(VI) concentration: 60 mg/L; contact time: 0.1, 0.25, 0.5, 0.75, 1, 1.5, 2 and 4 h; initial pH = 3.0; end pH: 2.70–2.90.

Figure 2 and Figure 3 show that U(VI) adsorption increased with rising temperature, indicating an endothermic process. This may be due to the increased binding sites of HA at a higher temperature [31]. Similar results were reported in the literature [8,25,31]. The time required for U(VI) adsorption process to reach equilibrium was 1.5 h at 298 K, 2 h at 308 K and 318 K.

Adsorption kinetics parameters are given in Table 2. The three models fit the adsorption process well (R^2^ > 0.95). The Elovich model had the highest R^2^, indicating that U(VI) adsorption onto HA may be chemisorption rather than intraparticle diffusion [18,20]. This was further evidenced by a low R^2^ value from the intraparticle diffusion equation (<0.70) in Table 3, which suggests that the adsorption process was not controlled by intraparticle diffusion.

### 4.3. Adsorption Isotherms

**Figure 4:**
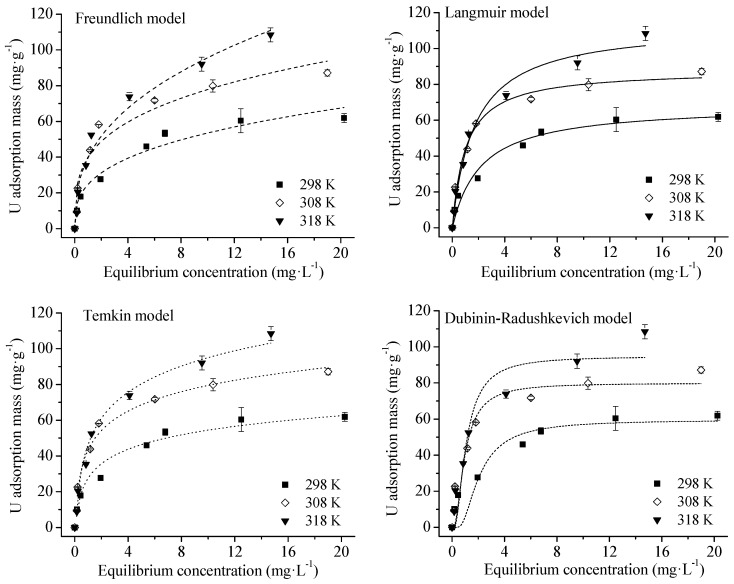
Isotherms of U(VI) adsorption onto HA at different temperatures. Experimental conditions: adsorbent mass: 20 mg; solution volume: 30 mL; U(VI) concentration: 0, 5, 10, 20, 40, 60, 80, 100 mg/L; contact time: 6 h; initial pH = 3.0; equilibrium pH: 2.60–2.90.

As shown in Figure 4, adsorption capacity increased with U concentrations. The parameters from fitting adsorption data into four isotherm models are given in Table 4.

The *n* values of Freundlich equation were higher than unity, indicating adsorption may be chemical rather than physical in nature with a high affinity of HA for U(VI), thus a high adsorption capacity [25,32]. Constant, *k_F_*, was related to adsorption capacity. Its increase with temperature also confirmed that U(VI) adsorption on HA was endothermic. Adsorption data fit the Langmuir model well (R^2^ > 0.95). The maximum adsorption capacity (*q_L_*) at the concentration range of 0–100 mg/L increased with temperature. Even at acidic condition (pH 3), *q_L_* of 68.6 mg·g^−1^ was higher than the adsorption capacities of common adsorbents (kaolin, biochar, activated carbon, hematite, and bentonite) at near-neutral pH that would not be observed in acidic effluents (Table 5). The large adsorption capacity of HA for U is in agreement with its abundant carboxyl group [13]. The good fit of experimental data with Temkin equation (R^2^ > 0.97) implied that U(VI) adsorption onto HA involved chemisorption [33]. This was further supported by the results of pseudo second-order and Elovich equations. The *q_D_* values of D–R model were not consistent with the *q_L_* calculated from the Langmuir isotherm as show in Figure 4 and Table 4. Fitting of adsorption data into the D–R model produced the lowest R^2^ in Table 4, further suggesting U(VI) adsorption onto HA was not a physical process [25,28,34,35].

### 4.4. Adsorption Thermodynamics

**Figure 5:**
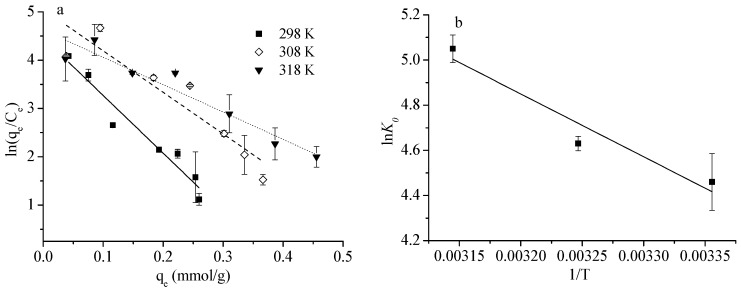
The calculation of thermodynamic parameters (**a**) ln*K_0_*, (**b**) Δ*H*^0^ and Δ*S*^0^.

The values of ln*K*_0_ at different temperatures were determined by linear plotting ln(q_e_/C_e_) versus q_e_, assuming q_e_ as zero as described in Figure 5a [8,14]. Δ*G*^0^ values were calculated from Equation (9) as displayed in Table 6. Δ*H*^0^ and Δ*S*^0^ were determined on the bases of Equation (10) by plotting ln*K*_0_ versus 1/T, included in Figure 5b. The negative Δ*G*^0^ indicated that the adsorption reaction was spontaneous, and its extent of spontaneity increased with rising temperature. A positive Δ*S*^0^ = 114.3 J·mol^−1^·K^−1^ suggested that U(VI) adsorption onto HA was endothermic, which was supported by the higher adsorption capacity at higher temperature. A positive Δ*H*^0^ = 23.13 kJ·mol^−1^ revealed that the HA had a high affinity for U(VI). Further, Δ*H*^0^ was a useful value to distinguish physisorption from chemisorption. In general, Δ*H*^0^ for physisorption is small, 2.1–20.9 kJ·mol^-1^, whereas Δ*H*^0^ for chemisorption is large, 20.9–418.4 kJ·mol^−1^ [38,39]. The value of Δ*H*^0^ in the range of 20.9–418.4 kJ·mol^−1^ indicated that the adsorption of U(VI) onto HA involved chemisorption [39].

### 4.5. Adsorption Mechanism

FTIR is a useful tool to probe adsorption behavior of cations onto adsorbents [8,23,40]. The vibration frequency changes in characteristic peaks of HA before and after adsorption (Figure 1) include the shifts of the symmetric -COO^−^ stretch frequency from 1601 to 1590 cm^−1^ (red shift), symmetric -COO^−^ stretch frequency from 1426 to 1416 cm^−1^ (red shift), salts of -COOH stretch frequency from 1368 to 1360 cm^−1^ (red shift), and phenolic C-OH stretch frequency from 1204 to 1219 cm^−1^ (blue shift). Thus, U(VI) reacted with HA through functional groups [8,41]. The FTIR analysis further elaborated that U(VI) adsorption onto HA was via chemisorption. The adsorption process could be controlled by surface or intraparticle diffusion, and the intraparticle diffusion model is often used to make the judgment [22,42]. The parameters and R^2^ of data fitting into the intraparticle diffusion model are given in Table 3. The low R^2^ (< 0.7) suggested that the adsorption process was not controlled by intraparticle diffusion. In other words, surface diffusion was the dominant process for U(VI) adsorption onto HA via chemisorption, such as ion-exchange, complexation and chelation [25,30].

### 4.6. The Effects of Cations and Anions on U(VI) Adsorption

**Figure 6:**
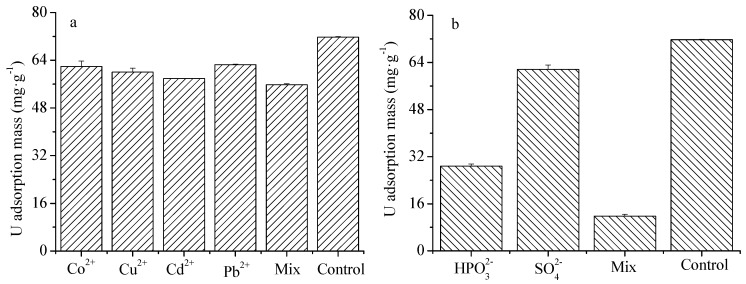
The effects of co-existing cations (**a**) or anions (**b**) on U(VI) adsorption onto HA. Experimental conditions: U(VI) concentration: 60 mg/L; single cation concentration: 10 mg/L; mix cation concentration: 40 mg/L; single anion concentration: 50 mg/L; mix anion concentration: 100 mg/L; contact time: 6 h; initial pH = 3.0; equilibrium pH = 2.70–2.90.

Anions and cations are common in acidic U contaminated water and in soil environment [43]. They may affect U(VI) adsorption onto HA. Figure 6 shows the effect of common cations and anions on U(VI) adsorption onto HA. The presence of Cu^2+^, Co^2+^, Cd^2+^ and Pb^2+^ cations reduced U(VI) adsorption capacity, which could be explained by the competitive adsorption of the cations for U(VI) [44,45]. However, they are not good competitors for U(VI) and Pb^2+^ was the least competitive. The U(VI) adsorption decreased as co-existing cation concentrations increased, which is consistent with previous studies [46,47]. The presence of anions HPO_4_^2−^ and SO_4_^2−^ greatly reduced the adsorption capacity of HA for U(VI) as shown in Figure 6b. For SO_4_^2−^, the reduced adsorption may be caused by the competition between SO_4_^2−^ and HA for UO_2_^2+^, or the formation of negatively charged complexes with UO_2_^2+^ [43,48,49]. At acidic condition, the HPO_4_^2−^ can react with H^+^ to form H_2_PO_4_^−^ and H_3_PO_4_ [50]. HPO_4_^2−^ had stronger effects than SO_4_^2−^. This may be caused by the formation of precipitation between UO_2_^2+^ and HPO_4_^2−^, H_2_PO_4_^−^ and H_3_PO_4_, which could prevent UO_2_^2+^ being adsorbed onto HA surface [48,50,51,52].

## 5. Conclusions

HA derived from leonardite was an effective adsorbent for removing uranium from aqueous acid solutions. The adsorption increased as temperature increased. Data fitting into kinetic models and large Δ*H*^0^ suggested that the adsorption involved chemisorption. The thermodynamic parameters indicated that the adsorption process was endothermic and spontaneous. Co-existing cations and anions had negative effects on U(VI) adsorption onto HA. Because of its wide availability and low-cost HA has a potential for use in the treatment of acidic mining effluents.

## Figures and Tables

**Table 1:** The selected properties of leonardite and leonardite-derived humic acid (HA).

Adsorbents	pH (H_2_O)	Ash (%)	C (%)	H (%)	N (%)	S (%)	O (%)	-COOH (mol/kg)	Phenolic-OH (mol/kg)
Leonardite	3.99	17.77	49.21	3.48	1.01	0.47	28.06	3.04	1.10
HA	2.78	7.48	56.71	3.78	1.16	0.36	30.51	3.64	1.03

**Table 2:** Parameters of kinetic models for U(VI) adsorption onto HA.

Temperature (*K*)	*q_e_* (mg·g^−1^)	Isotherm Model				
		Pseudo First-Order		Pseudo Second-Order		Elovich
		*q_1_* (mg·g^−1^)	R^2^	*q_2_* (mg·g^−1^)	R^2^	R^2^
298	28.60	27.58	0.986 **	28.86	0.997 **	0.986 **
308	29.96	28.36	0.971 **	29.99	0.994 **	0.991 **
318	34.79	33.56	0.989 **	34.89	0.999 **	0.992 **

*q_e_*: the measured adsorption mass at equilibrium state; *q_1_*: the adsorption mass calculated by pseudo first-order at equilibrium state; *q_2_*: the adsorption mass calculated by pseudo second-order at equilibrium state; ** Significant at 0.01 probability level.

**Table 3:** Intraparticle diffusion coefficients and intercept values for U(VI) adsorption on HA particles at different temperatures.

Temperature (*K*)	*k_i_* (mg·g^−1^·h^0.5^)	*C* (mg·g^−1^)	R^2^
298	3.99	22.50	0.553 **
308	5.17	21.89	0.684 **
318	4.24	28.21	0.599 **

*k_i_*: the intraparticle diffusion rate constant; *C*: a constant; ** Significant at 0.01 probability level.

**Table 4:** Parameters of adsorption isotherm models for U(VI) adsorption onto HA.

Isotherm	Parameters			R^2^
Freundlich model	Temperature (K)	*n*	*k_F_* (mg^(1−n)^·L^n^·g^−1^)	
	298	2.99	24.76	0.967 **
	308	3.44	39.88	0.940 **
	318	2.62	39.66	0.973 **
Langmuir model	Temperature (K)	*q_L_* (mg·g^−1^)	*k_L_* (L·mg^−1^)	R^2^
	298	68.60	0.46	0.970 **
	308	88.12	0.99	0.985 **
	318	113.34	0.59	0.982 **
Temkin model	Temperature (K)	*k_T_* (L·mg^−1^)	*b* (J·mol^−1^)	R^2^
	298	10.62	212.28	0.972 **
	308	15.93	163.39	0.988 **
	318	9.00	125.22	0.988 **
Dubinin–Radushkevich model	Temperature (K)	*q_D_* (mg·g^−1^)	*k_D_* (mol^2^·J^−2^)	R^2^
	298	59.55	2.29E−8	0.785 **
	308	79.93	6.32E−9	0.851 **
	318	94.74	6.89E−9	0.878 **

*n*: a constant related to adsorption intensity; *k_F_*: the equilibrium adsorption constant related to adsorption capacity; *q_L_* and *q_D_*: the theoretical maximum capacity; *k_L_*: a constant related to the affinity of the binding sites; *k_T_*: Temkin isotherm equilibrium binding constant; *b*: Temkin isotherm constant; *k_D_*: Dubinin–Radushkevich isotherm constant; ** Significant at 0.01 probability level.

**Table 5:** The comparison between this study and previous studies.

Materials	*C* (mg U^6+^/L)	pH	*q_m_* (mg/g)	References
HA	0−100	3.0	68.60	This study
Kaolin	20−80	5.0	4.52	[10]
Biochar	0−100	6.0	62.70	[11]
Activated Carbon	100−200	6.0	24.94	[9]
Hematite	0−100	-	3.36	[36]
Modified bentonite	100−600	6.0	29.6	[37]

*C*: the U^6+^ concentration range; The *q_m_* was calculated from the Langmuir equation.

**Table 6:** Thermodynamic parameters for U(VI) adsorption onto HA particles.

Temperature (K)	ln*K*_0_	Δ*G*^0^ (kJ·mol^−1^)
298	4.46 *	−11.1
308	4.63 **	−12.2
318	5.05 **	−12.9

* Significant at 0.05 probability level. ** Significant at 0.01 probability.

## References

[1] Rankin D.W. (2008). CRC Handbook of Chemistry and Physics.

[2] Campos M.B., de Azevedo H., Nascimento M.R.L., Roque C.V., Rodgher S. (2011). Environmental assessment of water from a uranium mine (Caldas, Minas Gerais State, Brazil) in a decommissioning operation. Environ. Earth Sci..

[3] Mudd G.M., Patterson J. (2010). Continuing pollution from the Rum Jungle U–Cu project: A critical evaluation of environmental monitoring and rehabilitation. Environ. Pollut..

[4] Xie S., Yang J., Chen C., Zhang X., Wang Q., Zhang C. (2008). Study on biosorption kinetics and thermodynamics of uranium by Citrobacter freudii. J. Environ. Radioactiv..

[5] U.S. Environmental Protection Agency (2012). Drinking Water Standards and Health Advisories.

[6] World Health Organization (2008). Guidelines for Drinking-Water Quality.

[7] Bhalara P.D., Punetha D., Balasubramanian K. (2014). A review of potential remediation techniques for uranium(VI) ion retrieval from contaminated aqueous environment. J. Environ. Chem. Eng..

[8] Meng F.D., Yuan G.D., Larson S.L., Ballard J.H., Waggoner C.A., Arslan Z., Han F.X. (2017). Removing uranium (VI) from aqueous solution with insoluble humic acid derived from leonardite. J. Environ. Radioactiv..

[9] D’Souza S.F., Sar P., Kazy S.K., Kubal B.S. (2006). Uranium sorption by Pseudomonas biomass immobilized in radiation polymerized polyacrylamide bio-beads. J. Environ. Sci. Heal. A.

[10] Haddad D., Mellah A., Nibou D., Khemaissia S. (2018). Promising enhancement in the removal of uranium ions by surface-modified activated carbons: Kinetic and equilibrium studies. J. Environ. Chem. Eng..

[11] Wang G., Wang X., Chai X., Liu J., Deng N. (2010). Adsorption of uranium (VI) from aqueous solution on calcined and acid-activated kaolin. Appl. Clay Sci..

[12] Zhang Z., Cao X., Liang P., Liu Y. (2013). Adsorption of uranium from aqueous solution using biochar produced by hydrothermal carbonization. J. Radioanal. Nucl. Ch..

[13] Soler-Rovira P., Madejón E., Madejón P., Plaza C. (2010). In situ remediation of metal contaminated soils with organic amendments: Role of humic acids in copper bioavailability. Chemosphere.

[14] Khalili F., Al-Banna C. (2015). Adsorption of uranium (VI) and thorium (IV) by insolubilized humic acid from Ajloun soil–Jordan. J. Environ. Radioactiv..

[15] Harter R.D., Naidu R. (2001). An assessment of environmental and solution parameter impact on trace-metal sorption by soils. Soil Sci. Soc. Am. J..

[16] International Humic Substances Society (2018). Acidic Functional Groups of IHSS Samples. http://humic-substances.org/acidic-functional-groups-of-ihss-samples/#products.

[17] Ho Y.S. (2004). Citation review of Lagergren kinetic rate equation on adsorption reactions. Scientometrics.

[18] Simonin J.P. (2016). On the comparison of pseudo-first order and pseudo-second order rate laws in the modeling of adsorption kinetics. Chem. Eng. J..

[19] Ho Y.S., McKay G. (1999). Pseudo-second-order model for sorption processes. Process. Biochem..

[20] Inyang H.I., Onwawoma A., Bae S. (2016). The Elovich equation as a predictor of lead and cadmium sorption rates on contaminant barrier minerals. Soil Till. Res..

[21] Juang R.S., Chen M.K. (1997). Application of the Elovich equation to the kinetics of metal sorption with solvent-impregnated resins. Ind. Eng. Chem. Res..

[22] Wu F.C., Tseng R.L., Juang R.S. (2009). Initial behavior of intraparticle diffusion model used in the description of adsorption kinetics. Chem. Eng. J..

[23] Meng F.D., Yuan G.D., Wei J., Bi D.X., Wang H.L. (2017). Leonardite-derived humic substances are great adsorbents for cadmium. Environ. Sci. Pollut. R..

[24] Yang C. (1998). Statistical mechanical study on the Freundlich isotherm equation. J. Colloid Interf. Sci..

[25] Boparai H.K., Joseph M., O’Carroll D.M. (2011). Kinetics and thermodynamics of cadmium ion removal by adsorption onto nano zerovalent iron particles. J. Hazard. Mater..

[26] Sadeek S.A., Negm N.A., Hefni H.H.H., Wahab M.M.A. (2015). Metal adsorption by agricultural biosorbents: Adsorption isotherm, kinetic and biosorbents chemical structures. Int. J. Biol. Macromol..

[27] Aharoni C., Ungarish M. (1977). Kinetics of activated chemisorption. Part 2—Theoretical models. J. Chem. Soc. Faraday Trans. 1.

[28] Celebi O., Kilikli A., Erten H.N. (2009). Sorption of radioactive cesium and barium ions onto solid humic acid. J. Hazard. Mater..

[29] Stevenson F.J. (1994). Humus Chemistry: Genesis, Composition, Reactions.

[30] Tan K.H. (2014). Humic Matter in Soil and the Environment: Principles and Controversies.

[31] Mahmoud M.A. (2016). Kinetics and thermodynamics of U (VI) ions from aqueous solution using oxide nanopowder. Process. Saf. Environ..

[32] Jiang J.Q., Zeng Z. (2003). Comparison of modified montmorillonite adsorbents: Part II: The effects of the type of raw clays and modification conditions on the adsorption performance. Chemosphere.

[33] Biswas K., Saha S.K., Ghosh U.C. (2007). Adsorption of fluoride from aqueous solution by a synthetic iron (III)−aluminum (III) mixed oxide. Ind. Eng. Chem. Res..

[34] Foo K.Y., Hameed B.H. (2010). Insights into the modeling of adsorption isotherm systems. Chem. Eng. J..

[35] Hutson N.D., Yang R.T. (1997). Theoretical basis for the Dubinin-Radushkevitch (DR) adsorption isotherm equation. Adsorption.

[36] Xie S., Zhang C., Zhou X., Yang J., Zhang X., Wang J. (2009). Removal of uranium (VI) from aqueous solution by adsorption of hematite. J. Environ. Radioactiv..

[37] Zareh M.M., Aldaher A., Hussein A.E.M., Mahfouz M.G., Soliman M. (2013). Uranium adsorption from a liquid waste using thermally and chemically modified bentonite. J. Radioanal. Nucl. Chem..

[38] Deng L., Su Y., Su H., Wang X., Zhu X. (2007). Sorption and desorption of lead (II) from wastewater by green algae Cladophora fascicularis. J. Hazard. Mater..

[39] Ma F., Qu R., Sun C., Wang C., Ji C., Zhang Y., Yin P. (2009). Adsorption behaviors of Hg (II) on chitosan functionalized by amino-terminated hyperbranched polyamidoamine polymers. J. Hazard. Mater..

[40] Zhu Q., Li Z. (2015). Hydrogel-supported nanosized hydrous manganese dioxide: Synthesis, characterization, and adsorption behavior study for Pb^2+^, Cu^2+^, Cd^2+^ and Ni^2+^ removal from water. Chem. Eng. J..

[41] Mukhopadhyay A., Pandey P., Chakraborty T. (2010). Blue-and red-shifting CH··O hydrogen bonded complexes between haloforms and ethers: Correlation of donor νC−H spectral shifts with C−O–C angular strain of the acceptors. J. Phys. Chem. A.

[42] Cheung W.H., Szeto Y.S., McKay G. (2007). Intraparticle diffusion processes during acid dye adsorption onto chitosan. Bioresource Technol..

[43] Wang P., Yin L., Wang J., Xu C., Liang Y., Yao W., Wang X., Yu S., Chen C., Sun Y. (2017). Superior immobilization of U (VI) and ^243^Am (III) on polyethyleneimine modified lamellar carbon nitride composite from water environment. Chem. Eng. J..

[44] Futalan C.M., Kan C.C., Dalida M.L., Hsien K.J., Pascua C., Wan M.W. (2011). Comparative and competitive adsorption of copper, lead, and nickel using chitosan immobilized on bentonite. Carbohyd. Polym..

[45] Park J.H., Ok Y.S., Kim S.H., Cho J.S., Heo J.S., Delaune R.D., Seo D.C. (2016). Competitive adsorption of heavy metals onto sesame straw biochar in aqueous solutions. Chemosphere.

[46] Feng Y., Gong J.L., Zeng G.M., Niu Q.Y., Zhang H.Y., Niu C.G., Deng J.H., Yan M. (2010). Adsorption of Cd(II) and Zn(II) from aqueous solutions using magnetic hydroxyapatite nanoparticles as adsorbents. Chem. Eng. J..

[47] Jiang M., Jin X., Lu X.Q., Chen Z. (2010). Adsorption of Pb(II), Cd(II), Ni(II) and Cu(II) onto natural kaolinite clay. Desalination.

[48] Bachmaf S., Planer-Friedrich B., Merkel B.J. (2008). Effect of sulfate, carbonate, and phosphate on the uranium (VI) sorption behavior onto bentonite. Radiochim. Acta.

[49] Lee S.Y., Cha W.S., Kim J.G., Baik M.H., Jung E.C., Jeong J.T., Kim K., Chung S.Y., Lee Y.J. (2014). Uranium (IV) remobilization under sulfate reducing conditions. Chem. Geol..

[50] Sanding A., Bruno J. (1992). The solubility of (UO_2_)_3_(PO_4_)_2_•4H_2_O (s) and the formation of U(VI) phosphate complexes: Their influence in uranium speciation in natural waters. Geochim. Cosmochim. Acta.

[51] Mehta V.S., Maillot F., Wang Z., Catalano J.G., Giammar D.E. (2014). Effect of co-solutes on the products and solubility of uranium (VI) precipitated with phosphate. Chem. Geol..

[52] Perassi I., Borgnino L. (2014). Adsorption and surface precipitation of phosphate onto CaCO_3_–montmorillonite: Effect of pH, ionic strength and competition with humic acid. Geoderma.

